# Natural product biosyntheses in cyanobacteria: A treasure trove of unique enzymes

**DOI:** 10.3762/bjoc.7.191

**Published:** 2011-12-05

**Authors:** Jan-Christoph Kehr, Douglas Gatte Picchi, Elke Dittmann

**Affiliations:** 1 University of Potsdam, Institute for Biochemistry and Biology, Karl-Liebknecht-Str. 24/25, 14476 Potsdam-Golm, Germany

**Keywords:** cyanobacteria, natural products, NRPS, PKS, ribosomal peptides

## Abstract

Cyanobacteria are prolific producers of natural products. Investigations into the biochemistry responsible for the formation of these compounds have revealed fascinating mechanisms that are not, or only rarely, found in other microorganisms. In this article, we survey the biosynthetic pathways of cyanobacteria isolated from freshwater, marine and terrestrial habitats. We especially emphasize modular nonribosomal peptide synthetase (NRPS) and polyketide synthase (PKS) pathways and highlight the unique enzyme mechanisms that were elucidated or can be anticipated for the individual products. We further include ribosomal natural products and UV-absorbing pigments from cyanobacteria. Mechanistic insights obtained from the biochemical studies of cyanobacterial pathways can inspire the development of concepts for the design of bioactive compounds by synthetic-biology approaches in the future.

## Introduction

### The role of cyanobacteria in natural product research

**Figure 1:**
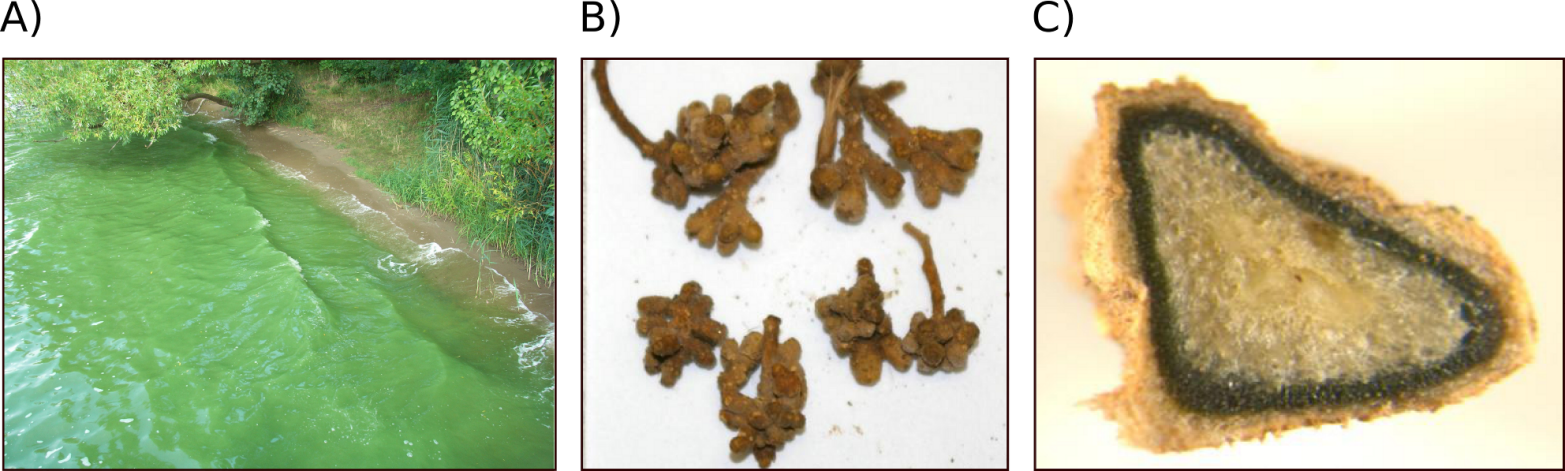
Cyanobacteria proliferate in diverse habitats. A) Bloom-forming freshwater cyanobacteria of the genus *Microcystis*. B) Roots of cyanobacterial symbiosis host *Cycas circinalis*. C) Terrestrial cyanobacteria living in corraloid roots of *Cycas circinalis*.

Cyanobacteria flourish in diverse ecosystems and play an enormous role in the biogeochemical cycles on earth. They are found in marine, freshwater and terrestrial environments and even populate such extreme habitats as the Antarctic or hot springs [1]. Due to their capability to fix nitrogen from the atmosphere some species are attractive partners in symbioses [2] (Figure 1). Other cyanobacteria show a strong tendency for mass developments during summer months, so-called blooms [3] (Figure 1). Cyanobacteria do not belong to the established sources of natural products and are only incidentally screened by pharmaceutical industries. For a long time, natural product research on cyanobacteria was mostly focused on toxins, in particular on the widespread hepatotoxin microcystin **1** [4]. Starting in the eighties, however, a number of promising compounds was isolated by means of bioactivity-guided screening techniques towards cytotoxic, multidrug-resistance reversal, antiprotease, antifungal and antiviral activities [5]. Many bioactive metabolites possess a peptide or a macrolide structure, or a combination of both types [6–8]. Other metabolites belong to the alkaloid class of compounds. In the last two decades, biosynthesis gene clusters were assigned to an increasing number of these cyanobacterial natural products [7,9]. Part of the genetic analyses was assisted by biochemical studies of the enzymes. These studies revealed a truly fascinating variety of enzymatic features, including many that are not or only rarely seen in other microorganisms. The potential of cyanobacteria for natural product research thus goes far beyond the exploitation of the bioactivity of the products. Knowledge about the biochemistry of unique enzymes is particularly valuable for synthetic biology approaches towards libraries of new compounds or for rational biotransformation of existing leading compounds. This review gives an overview of the current trends in cyanobacterial natural-product research, with a special emphasis on the biosynthetic enzymes.

## Review

### Biosynthesis of peptides and polyketides in microorganisms

**Figure 2:**
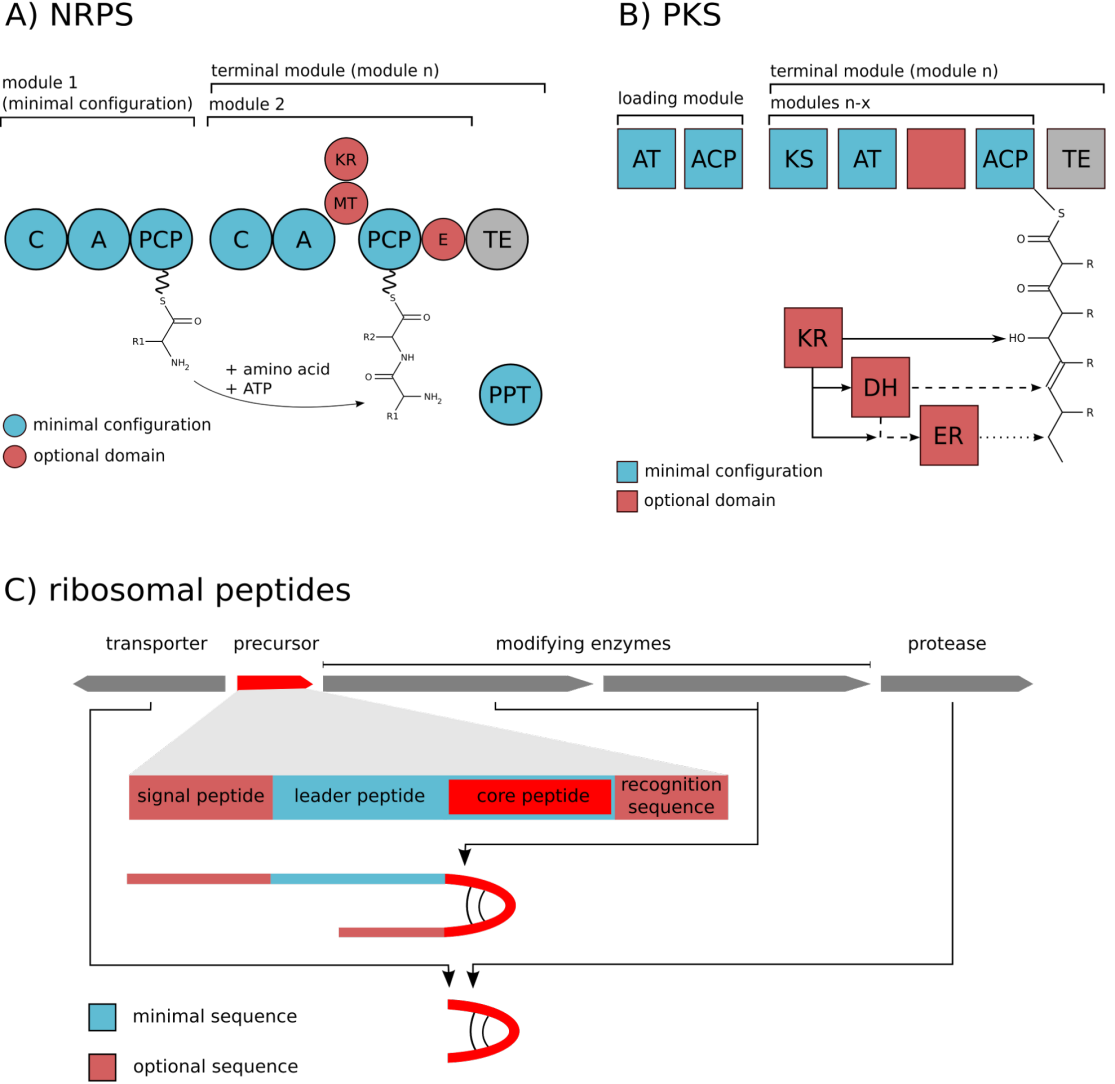
Schematic representation of enzymatic domains in A) nonribosomal peptide synthetases (NRPS); B) polyketide synthases (PKS) and C) the typical organisation of a ribosomal biosynthetic gene cluster. Abbreviations: C: Condensation domain; A: Adenylation domain; PCP: Peptidyl carrier protein; MT: Methyltransferase; E: Epimerase; AT: Acyltransferase; ACP: Acyl carrier protein; KS: Ketosynthase; KR: Ketoreductase; DH: Dehydratase; ER: Enoyl reductase; TE: Thioesterase.

Microbial natural products of the peptide class are produced by two types of biosynthetic pathways: By giant multi-domain enzymes, the nonribosomal peptide synthetases (NRPS) or by ribosomal synthesis and subsequent post-translational modification and processing. NRPS consist of modules, each being responsible for the incorporation of a single amino acid. The order of these modules typically follows a colinearity rule, i.e., the succession of modules corresponds to the order of amino acids in the final product. A minimal module is composed of an amino acid-activating adenylation (A) domain, a peptidyl carrier (PCP) domain carrying the phosphopantetheine cofactor, and a condensation (C) domain (Figure 2) [10]. NRPS can accept about 300 proteinogenic and nonproteinogenic substrates and may contain further domains introducing tailoring modifications or epimerizing the amino acid substrates [11]. In contrast, ribosomal biosynthesis of peptides is limited to 20 proteinogenic amino acids. This group of peptides nevertheless displays a high diversity and a considerable biosynthetic and bioactive potential. The ribosomal prepeptides are typically composed of a leader peptide and a core peptide. Associated post-translational modification enzymes (PTMs) catalyze different types of macrocyclizations of the core peptide and side-chain modifications of amino acids. Peptide maturation further requires cleavage of the leader peptide by processing proteases (PP) frequently combined with transport across the plasma membrane [12] (Figure 2).

Macrolides in microorganisms are produced by modular type polyketide synthases (PKS) resembling NRPS with respect to their modular nature. In contrast to the peptide-synthesizing enzymes, different types of carboxylic acids are activated, assembled and optionally modified. The maximal set of domains of an individual PKS module is identical to animal fatty acid synthase (FAS) [13] and consists of ketosynthase (KS), acyltransferase (AT), ketoreductase (KR), dehydratase (DH), enoyl reductase (ER) and acyl carrier protein (ACP) domains [14]. Parts of the domains (KR, DH, ER) are optionally used leading to a different reduction state of the keto groups of polyketides. There are also alternative PKS assembly lines cooperating with AT domains encoded in trans of the multienzymes [15], or PKS types comprising single modules that work iteratively [16].

### Nonribosomal peptide, polyketide and hybrid biosyntheses in cyanobacteria

Research on NRPS and PKS gene clusters started almost in parallel in freshwater, marine and terrestrial cyanobacteria. A major trait of cyanobacterial pathways is their hybrid character, i.e., the frequent mixture of NRPS and PKS modules. Hereafter, we highlight the most interesting biochemical features of cyanobacterial assembly lines. Although we specifically refer to enzymes that were analyzed biochemically, the additional unusual characteristics of the compounds (the formations of which remain to be elucidated) will be mentioned. The list is divided into freshwater, marine and terrestrial biosyntheses and arranged chronologically according to the first description of the respective biosynthetic pathway. As cyanobacterial secondary metabolites frequently occur as classes of related molecules, only a single representative of each structural class will be discussed.

### NRPS and PKS pathways in freshwater cyanobacteria

#### Microcystin

**Figure 3:**
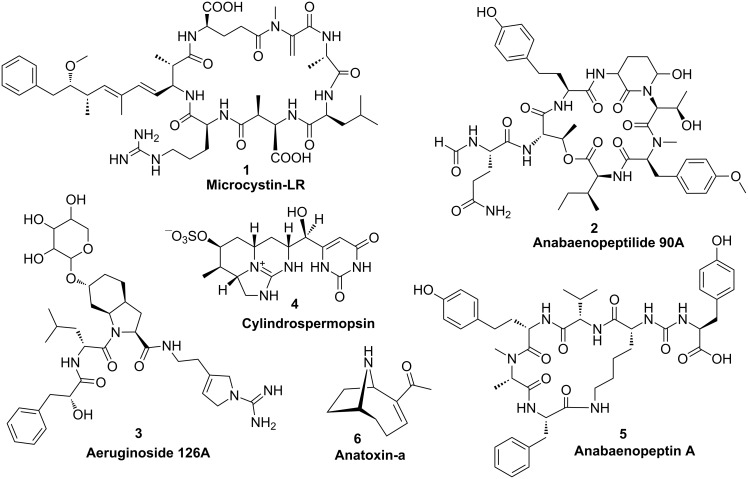
Structures of NRPS and PKS products in freshwater cyanobacteria.

**Figure 4:**
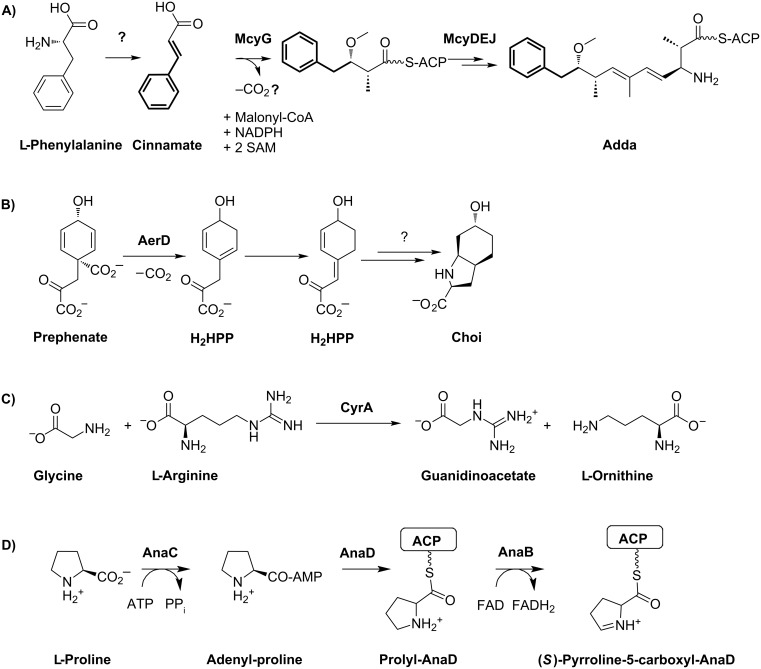
A) Synthesis of the Adda ((2*S*,3*S*,8*S*,9*S*)-3-amino-9-methoxy-2,6,8-trimethyl-10-phenyl-4,6-decadienoic acid) moiety of microcystin (**1**) starting with cinnamate. The mechanism of α-carbon decarboxylation has to be elucidated. B) Synthesis of the Choi moiety of the aeruginosin **3**. H_2_HPP: Dihydro-4-hydroxyphenylpyruvate. C) Formation of the guanidinoacetate starter unit for the subsequent PKS assembly line of cylindrospermopsin (**4**). D) Formation of the (*S*)-pyrroline-5-carboxylate starter unit from proline in anatoxin-a (**6**) synthesis. ACP: Acyl carrier protein.

The first biosynthetic pathway identified and partially characterized for cyanobacteria was the mixed NRPS/PKS pathway catalyzing the formation of the hepatotoxin microcystin **1** (Figure 3) in the cyanobacterium *Microcystis aeruginosa* [17–19]. Microcystins are produced by different genera of freshwater cyanobacteria and inhibit eukaryotic protein phosphatases of types 1 and 2A. A signature of this heptapeptide family is the unusual β-amino acid Adda (3-amino-9-methoxy-2,6,8-trimethyl-10-phenyldeca-4,6-dienoic acid); more than 60 isoforms of the peptide have been described. The pentapeptide nodularin is structurally closely related and shares a highly similar biosynthetic pathway [20]. The biosynthetic assembly line of microcystin was predicted to start with the activation of phenylacetate; however, in vitro studies showed that phenylpropanoic acids are preferentially activated and loaded onto the neighboring PCP carrier domain and disproved the activation of phenylacetate [21]. Nevertheless, in order to generate the expected polyketide chain one carbon must be excised following extension with malonyl-CoA. The mechanism by which this truncation occurs is currently unknown (Figure 4A). Further unique features of microcystin biosynthesis include the standalone aspartate racemase McyF [22], the *O*-methyltransferase McyJ [23] and the 2-hydroxy-acid dehydrogenase McyI [24]. In addition, microcystins contain two amino acids that are linked with their ω-carboxy group in the peptide chain, namely glutamate and aspartate. Although the mechanism by which these amino acids are activated is still unknown, the microcystin biosynthesis pathway exemplifies the high number of unique features in cyanobacterial modular biosyntheses.

#### Anabaenopeptilide

The anabaenopeptilide pathway in the strain *Anabaena* 90 was described soon after first reports about microcystin biosynthesis. Anabaenopeptilides **2** belong to the cyanopeptolin family of depsipeptides that were shown to inhibit different types of serine proteases [7]. Like microcystins, these peptides are frequently produced by bloom-forming freshwater cyanobacteria. The signature of this group is the unusual 3-amino-6-hydroxy-2-piperidone moiety (Ahp). The corresponding NRPS assembly line consists of seven modules [25]. Unique features include an integrated formyl transferase domain in the initiation module and NAD-dependent halogenase. The formation of Ahp remains to be analyzed.

#### Aeruginosin

Aeruginosins are specific inhibitors of serine type proteases and produced by different genera of freshwater cyanobacteria. The strain *Planktothrix agardhii* NIVA-CYA 126 was used to identify and partially characterize the corresponding biosynthetic pathway [26]. The strain produces glycosylated variants of the peptides, aeruginosides **3**, via a mixed NRPS/PKS pathway. The signature of this group is the 2-carboxy-6-hydroxyoctahydroindole (Choi) moiety. The loading module was predicted to activate phenylpyruvate which is reduced by an integrated KR domain to phenyllactate [26]. Mutational analyses have revealed that the Choi moiety is synthesized by the three enzymes AerD, AerE and AerF [26]. AerD has been shown to catalyze a prephenate decarboxylation step [27], the exact roles of AerE and AerF remain to be elucidated (Figure 4B). The succeeding NRPS adenylation domain is then directly activating Choi as a substrate. The aeruginoside assembly line does not contain a thioesterase or reductase domain [26]. It is thus currently unclear how the final product is released from the enzyme complex.

#### Cylindrospermopsin

Cylindrospermopsin (**4**) is a hepatotoxin produced by different genera of freshwater cyanobacteria, including *Cylindrospermopsis raciborskii*, *Aphanizomenon ovalisporum* and *Aphanizomenon flos-aquae*. The polyketide-derived alkaloid inhibits glutathione and protein synthesis as well as cytochrome P450. Characteristic features of cylindrospermopsins include a guanidine moiety and a hydroxymethyluracil attached to the tricyclic carbon skeleton. According to feeding assays, the polyketide chain assembly starts with the activation of guanidinoacetate [28]. The precursor is formed via the activity of the unique L-arginine-glycine amidinotransferase CyrA (AoaA in *A. ovalisporum*) [29–30] (Figure 4C). The assembly line further comprises seven additional malonyl-CoA specific PKS modules [28]. It has been discussed that the three cyclization steps necessary for the formation of the characteristic tricyclic structure of cylindrospermopsins occur spontaneously by Michael addition during polyketide elongation rather than by enzymatic control [28]. Two enzymes, CyrG and CyrH with similarity to amidohydrolases/ureases/dihydroorotases are discussed to be responsible for uracil ring formation, although biochemical evidence is currently missing. The final hydroxylation step towards cylindrospermopsin has been shown to be catalyzed by CyrI, a 2-oxoglutarate-dependent iron oxygenase [31]. Interestingly, two epimers were described for the corresponding hydroxyl group resulting in either 7-*epi*-cylindrospermopsin or cylindrospermopsin. The proportion of these two epimers varies in different cylindrospermopsin producing strains. It remains to be shown if the corresponding hydroxylase produces both stereoisomers or if a second unidentified hydroxylase is involved in the alternative epimer formation [32]. The CyrJ protein is the candidate protein for the sulfatation tailoring step.

#### Anabaenopeptin

Anabaenopeptins **5** are a highly diverse family of cyclic hexapeptides produced by various genera of freshwater cyanobacteria. Several members of the family potently inhibit proteases. A signature of the group is the conserved ureido linkage connecting the side-chain amino acid to D-lysine. The corresponding NRPS gene cluster was first analyzed in the strain *Anabaena* sp. 90 and revealed a new mechanism underlying production of diverse variants by the same strain: Two alternative NRPS starter modules [33]. Other cyanobacterial strains achieve diversity of anabaenopeptins by a different mode: Promiscuous A-domains [34]. The anabaenopeptin cluster contains five additional NRPS modules and an uncharacterized protein with similarity to pyruvate carboxyltransferases. The mechanism of ureido bond formation remains to be elucidated. Recently, ureido bond formation was characterized for the protease inhibitor syringolin A that is produced by *Pseudomonas syringiae* [35]. The responsible freestanding NRPS module contains a sequence stretch with similarity to acyltransferases between the condensation and adenylation domain. This sequence stretch does not show homology to any of the anabaenopeptin (Apt) biosynthesis proteins, suggesting a different mechanism of ureido bond formation for anabaenopeptins and syringolin [33].

#### Anatoxin

Anatoxin-a (**6**) and homoanatoxin-a are potent neurotoxins produced by cyanobacteria. A gene cluster for the alkaloid was first described for the strain *Oscillatoria* sp. PCC 6506 [36]. Analysis of the gene cluster and feeding studies suggested a biosynthetic scheme starting from L-proline and involving three polyketide synthases, with (*S*)-1-pyrolline-5-carboxylate proposed as the starter (Figure 4D) [37]. The first part of the biosynthesis could be reproduced with the acyl carrier protein AnaD, the Sfp-like phosphopantetheinyl transferase OsPPT, the A domain protein AnaC and the prolyl-AnaD dehydrogenase AnaB. The resulting (*S*)-pyrroline-5-carboxyl-AnaD is assumed to be the starter of polyketide chain assembly at the polyketide synthase AnaE [38]. The following polyketide extension step is predicted to be catalyzed by the polyketide synthase AnaF. The predicted protein ORF1 that is encoded in direct proximity of the *ana* cluster is expected to catalyze a Claisen-type cyclization step to form the characteristic bicyclic ring structure of anatoxin while the growing chain is tethered to the AnaF ACP domain. Experimental evidence for this suggestion is currently lacking. Finally, the bicyclic thioester is suggested to be transferred to the polyketide synthase AnaG for chain extension and is followed by chain release, which is expected to be catalyzed by the type II thioesterase AnaA. The reaction scheme as proposed would yield 11-carboxyanatoxin-a and 11-carboxyhomoanatoxin-a. Either a spontaneous or an enzymatically catalyzed decarboxylation step is thus necessary to finally yield anatoxin-a and homoanatoxin-a [38].

### NRPS and PKS pathways in marine cyanobacteria

#### Barbamide

**Figure 5:**
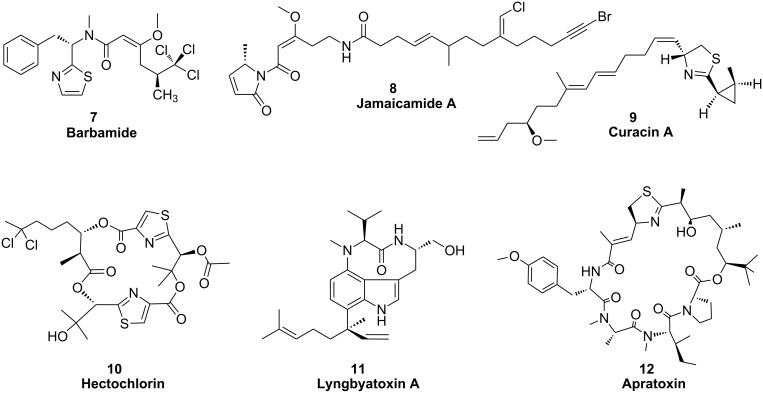
Structures of NRPS and PKS products in marine cyanobacteria.

**Figure 6:**
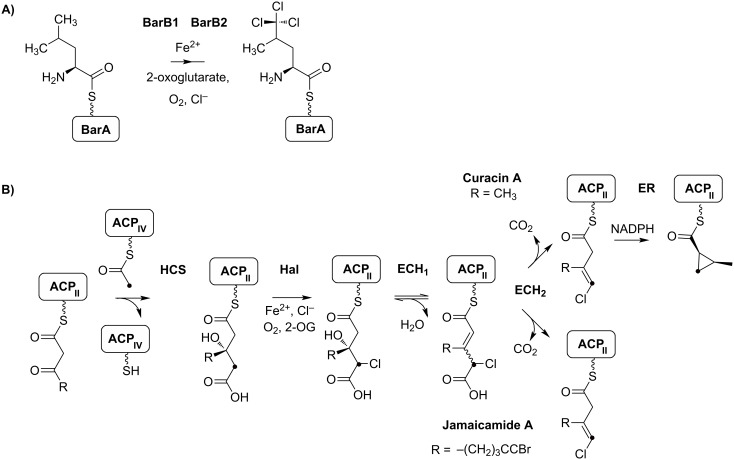
A) Formation of the trichloroleucyl starter unit of barbamide (**7**) synthesis through the non-heme iron(II)-dependent halogenases BarB1 and BarB2. B) Formation of cyclopropane and vinyl chloride functional groups in curacin A (**9**) and jamaicamide A (**8**) biosynthesis, respectively. The halogenated carbon is highlighted with a black dot. ACP: Acyl carrier protein; HCS: HMG-CoA synthase-like enzyme; Hal: Halogenase; ECH_1_: Dehydratase; ECH_2_: Decarboxylase; ER: Enoyl reductase.

Several NRPS/PKS assembly lines were identified and partially characterized for the marine cyanobacterium *Lyngbya majuscula* [39]. These filamentous tropical cyanobacteria are important contributors to coral reef ecosystems and extremely rich in bioactive secondary metabolites. The first pathway described was the biosynthesis of barbamide (**7**) (Figure 5), a chlorinated lipopeptide with potent molluscidal activity. The lipopeptide contains a unique trichloroleucyl starter unit that is halogenated by unique biochemical mechanisms through the two non-heme iron(II)-dependent halogenases BarB1 and BarB2 (Figure 6A) [40]. Further extraordinary features of the pathway include one-carbon truncation during chain elongation, *E*-double bond formation and thiazole ring formation.

#### Jamaicamide

The second biosynthetic pathway identified in a strain of the tropical marine cyanobacterium *Lyngbya majuscula* was assigned to jamaicamides **8**. Jamaicamides are neurotoxins and show sodium-channel-blocking activity. The lipopeptide is highly functionalized and contains a bromo-alkynyl, a chlorovinylidene substituent, a beta-methoxy eneone system, and a pyrrolinone ring [41]. The incorporation of the chlorovinylidene group was partially elucidated and predicted to be highly similar to the cyclopropane ring formation of curacin A biosynthesis (Figure 6B) ([42], see below). The enzyme cassette comprises a PKS module containing an integrated halogenase and a tandem acyl carrier protein tridomain (ACP_3_), a discrete ACP_IV_, a discrete ketosynthase (KS), a 3-hydroxy-3-methylglutaryl CoA synthetase (HMGCS), a dehydratase (ECH_1_)/decarboxylase (ECH_2_) pair and an enoyl reductase (ER) domain. HMG-CoA synthetases were shown to introduce β-branching carboxyl units into a number of polyketide chains. Feeding studies for jamaicamides revealed the incorporation of an acetate unit at the corresponding position followed by dehydratisation, decarboxylation and halogenation [41]. Further interesting features of jamaicamide biosynthesis include a six-carbon carboxylic acid unit as starter moiety. In vitro studies revealed the activation of either hexanoic, hexenoic or hexynoic acids at the JamA enzyme, whereas bromination clearly succeeded thioester formation [43].

#### Curacin A

Curacin A (**9**) was originally isolated from a *Lyngbya majuscula* strain found in Curacao, and it exhibits potent antiproliferative and cytotoxic activities [44]. This intriguing structure contains a thiazoline and a cyclopropyl ring. Interestingly, the pathway comprises a HMG-CoA synthase cassette, highly similar to the one of the jamaicamide assembly line, including the PKS module with the tandem acyl carrier protein tridomain (ACP_3_) as well as the discrete ACP_IV_, the discrete ketosynthase, the 3-hydroxy-3-methylglutaryl CoA synthetase (HMGCS), the dehydratase (ECH_1_)/decarboxylase (ECH_2_) pair and the ER domain [42,44]. Notably, the PKS module harbors a halogenation domain, although this could not be expected from the structure of curacin A. In vitro studies revealed that indeed cyclopropyl ring formation is preceded by a halogenation step (Figure 6B) [42]. The Cur ECH_2_ was found to catalyze the formation of a α,β-enoyl thioester, which is in contrast to the related enzyme of the Jam pathway, which generates a β,γ-thioester of the 3-methyl-4-chloroglutaconyl decarboxylation intermediate product [42]. The jamaicamide and curacin pathways thus provide a nice example of how diversification of single enzymes can lead to very different functionalities in the product.

The initiation module of curacin biosynthesis contains a GCN5-related *N*-acetyltransferase (GNAT) domain. These enzymes typically catalyze acyl transfer to a primary amine. The curacin GNAT, however, was shown to be bifunctional and to exhibit decarboxylase/*S*-acetyltransferase activities [45]. The corresponding PKS module was found to activate malonyl-CoA, which is expected to be transferred to the thiol group of the adjacent ACP domain via the embedded GNAT domain. However, only an acetyl group could be detected on the ACP, supporting an additional decarboxylase activity for the GNAT domain [46].

#### Hectochlorin

Hectochlorin (**10**) was isolated from a Jamaican isolate of *Lyngbya majuscula* and exhibits antifungal activity. It also shows potent activity towards a number of cancer cell lines. HctA, an acyl-CoA synthetase homologue, is expected to activate free hexanoic acid and to provide the starter for hectochlorin synthesis. Chain elongation is performed by a monomodular PKS and two bimodular NRPS. Two of the NRPS modules are suggested to activate 2-oxo-isovaleric acid, which is reduced by an embedded KR domain to 2-hydroxyisovaleric acid and proposed to be further oxidized by one of two cytochrome-P450-type monooxygenases encoded by the cluster, HctG or HctH, to 2,3-dihydroxyisovaleric acid. Two other NRPS modules contain all the required domains for adenylation and heterocyclization of cysteine, and an FMN-dependent oxidase domain, which is likely involved in thiazole ring formation. The cluster further encodes a halogenase/ACP didomain protein that is suggested to be responsible for the gem-dichloro group in hectochlorin, HctB. The specifities of the NRPS adenylation domains have been confirmed in vitro [47].

#### Lyngbyatoxin

Lyngbyatoxins **11**, produced by *Lyngbya majuscula,* can cause skin irritations and are implicated in the so-called “swimmers itch”. The compounds are also potent tumor promoters, which operate by competitively binding to protein kinase C (PKC). The characteristic indolactam ring of the toxin is synthesized by the bimodular NRPS LtxA [48]. The resulting dipeptide is tethered to a PCP domain and reductively released by the terminal NADPH-dependent reductase domain of LtxA [49]. The indolactam ring formation further requires the activity of the P450-dependent monooxygenase/cyclase LtxB [50]. Finally, the *ltx* cluster encodes the aromatic prenyltransferase LtxC, which was shown to catalyze geranyl pyrophosphate (GPP) addition to the indolactam ring in vitro [48].

#### Apratoxin

Apratoxin A (**12**) was isolated from a *Lyngbya bouillonii* strain isolated from a shallow-reef environment surrounding the island of Guam, Palau. Apratoxin A is a potent cytotoxin showing selective toxicity to cancer cells grown on solid agar as well as in the case of in vivo mouse models [51]. The corresponding hybrid PKS/NRPS pathway was identified through a single-cell genome-amplification approach and features a PKS-type loading module and nine extension modules (four PKS and five NRPS) [52]. Unique features of the cluster include a putative GCN_5_-related transferase, which is suggested to transfer three methyl groups from *S*-adenosyl-methionine (SAM) to malonyl-CoA to yield the *tert*-butyl terminus of apratoxin. The cluster further encodes a series of proteins resembling an HCS-like gene cassette that is expected to be involved in β-branching of polyketides [52].

### NRPS and PKS pathways in terrestrial cyanobacteria

#### Nostopeptolide

**Figure 7:**
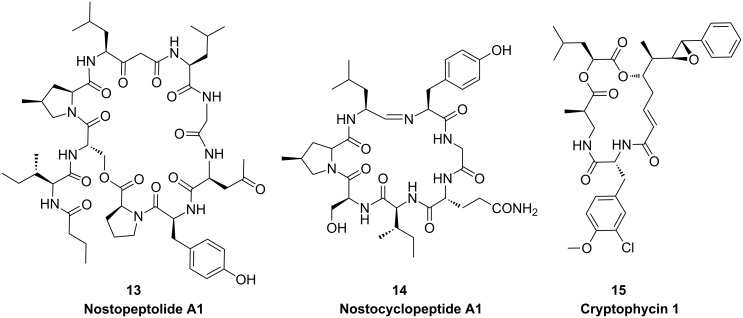
Structures of NRPS and PKS products in terrestrial cyanobacteria.

**Figure 8:**
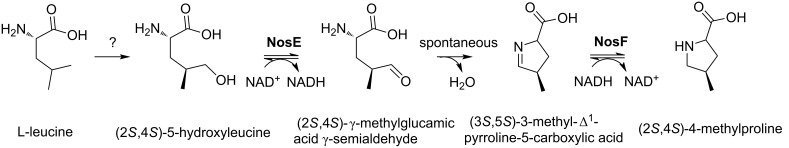
Synthesis of the (2*S*,4*S*)-4-methylproline moiety of nostopeptolides A (**13**).

The nostopeptolide gene cluster was the first NRPS/PKS type gene cluster described for a terrestrial cyanobacterial strain, namely *Nostoc* sp. GSV 224 [53]. Nostopeptolides **13** (Figure 7) are nonapeptides carrying a butyric acid and an internal acetate-derived unit. No cytotoxic, antifungal or protease-inhibition activities could be assigned to the compound. One of the unique components of the peptide backbone is the nonproteinogenic amino acid L-4-methylproline, which is synthesized from L-leucine by the zinc-dependent long-chain dehydrogenase NosE and the Δ^1^-pyrroline-5-carboxylic acid reductase homologue NosF (Figure 8) [54].

#### Nostocyclopeptide

The nostocyclopeptide **14** pathway is one of the few pure NRPS assembly lines of cyanobacteria and has been described for the terrestrial cyanobacterium *Nostoc* sp. ATCC 53789 [55]. The cyclic heptapeptide shares the L-4-methylproline unit of nostopeptolides and is synthesized by two enzymes closely resembling NosE and NosF. A unique feature of nostocyclopeptide biosynthesis is the mechanism of macrocyclization through imino bond formation between the N-terminal and C-terminal amino acids. The responsible enzyme NcpB contains a C-terminal reductase domain that has been shown to catalyze the reductive release of the peptide from the synthetase as an aldehyde followed by spontaneous formation of the imino head-to-tail linkage [56].

#### Cryptophycin

Cryptophycins were shown to be produced by terrestrial strains of *Nostoc* that are either free living or associated with a lichen symbiont. Cryptophycin 1 (**15**) is the most potent tubulin-destabilizing compound ever discovered and serves as a leading product for the development of cancer therapeutics. The corresponding biosynthetic pathway comprises three PKS and two NRPS-type enzymes [57]. The chain-initiation module of the enzyme CrpA closely resembles the loading module of microcystin biosynthesis where phenylpropanoic acids are activated and finally phenylacetate is incorporated in the product [21]. Characteristic features of the pathway further include an adenylation/ketoreductase didomain for the generation of α-hydroxy acids following activation of leucine [57]. The pathway also features an FAD-dependent halogenase [57] and the CYP450 epoxidase CrpE [58].

### Peptides ribosomally produced and post-translationally modified

**Figure 9:**
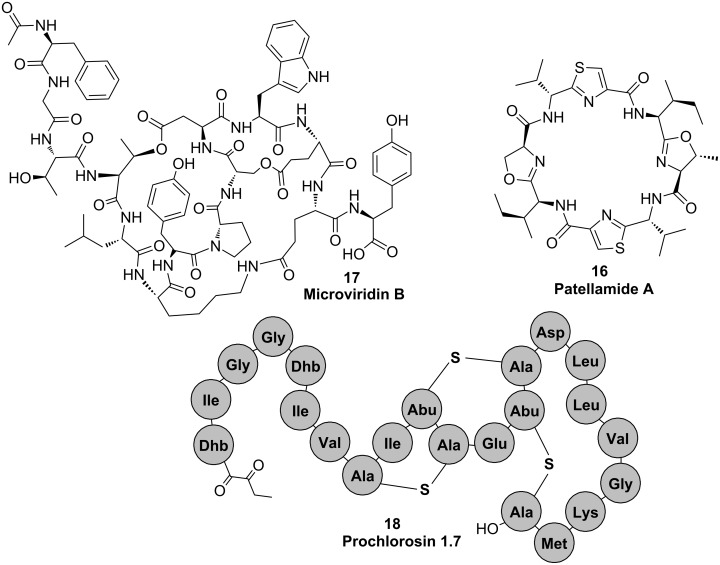
Structures of cyanobacterial peptides that are synthesized ribosomally and post-translationally modified.

Although the majority of cyanobacterial peptides are produced nonribosomally, for two peptide families, namely patellamides **16** and microviridins **17** (Figure 9), no NRPS pathway could be assigned, thus initiating research on ribosomal peptide pathways in cyanobacteria. Genome-scale analyses have unravelled further peptide families. Cyanobacteria can now be considered as one of the most prolific sources of ribosomally produced natural products. Hereafter, we give an overview of three major peptide families and their underlying biochemistry.

#### Cyanobactins

The first ribosomal pathway discovered was the biosynthesis of patellamides in the symbiotic cyanobacterium *Prochloron*. The cyclic octapeptides are pseudosymmetric and contain thiazole and oxazolin rings. Patellamides **16** are typically moderately cytotoxic, and some variants were further reported to reverse multidrug resistance [59]. The patellamide gene cluster consists of seven genes, expression of which in *E. coli* leads to the production of the peptides [59]. Heterocyclization of serine, cysteine and threonine, respectively is catalyzed by the heterocyclase PatD [60]. In contrast to other heterocyclases studied, PatD is a single ATP-dependent enzyme not requiring an additional oxidase enzyme [60]. The PatG protease encoded by the cluster was shown to macrocyclize diverse substrates by a mechanism closely resembling the thioesterase-catalyzed chemistry of most NRPS systems [61]. Several related pathways were later discovered in a variety of cyanobacterial strains. The PatG-type of macrocylization is the signature of this diverse group, which is now called the cyanobactin family [62–63]. The pathways optionally contain prenyltransferases: The substrate specificity and scope of which remain to be determined [62–63].

#### Microviridins

**Figure 10:**
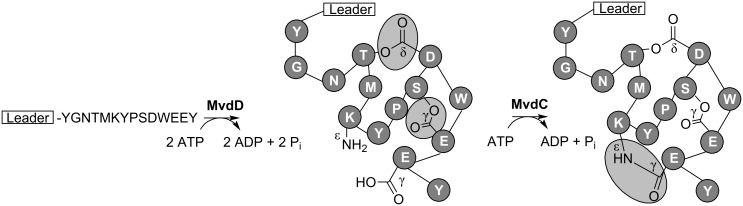
Formation of ester linkages and ω-amide linkage in microviridins **17** by the ATP grasp ligases MvdD and MvdC, respectively.

Microviridins **17** are a group of tricyclic depsipeptides predominantly detected in bloom-forming freshwater cyanobacteria. Several members of the family potently inhibit various serine-type proteases. The biosynthetic pathway of microviridins was described for the genera *Microcystis* and *Planktothrix* [64–65]. Post-translational modification of microviridins is achieved by the activity of two closely related ATP grasp ligases, MdnB and MdnC (MvdC and D in *Planktothrix*). The enzymes introduce two ω-ester linkages between threonine and aspartate and serine and glutamate (MdnC/MvdD) and one ω-amide linkage between lysine and aspartate (MdnB/MvdC) (Figure 10) [64–65]. Microviridins can be heterologously produced in *E. coli* [65]. Cyclizations occur in a strictly defined order. Ring size and composition of the microviridin core peptide is invariant [66], whereas N-terminal and C-terminal amino acids are highly variant [67]. The enzyme system further contains a GNAT-type *N*-acetyltransferase and an ABC transporter. The mechanism, by which the leader peptide of microviridins is cleaved off, remains to be elucidated.

#### Lantipeptides

Lantipeptides are produced by various types of bacteria. The characteristic feature of the group is lanthionine bridges, which are formed by dehydration of serine or threonine followed by intramolecular addition of cysteine thiols to the resulting dehydro amino acids. Lantipeptides exhibit a variety of bioactivities, in particular antimicrobial activities (lantibiotics) [68]. Cyanobacteria were shown to frequently encode LanM type enzymes, i.e., bifunctional enzymes catalyzing both dehydration and cyclization reactions [68]. An interesting phenomenon was observed for the strain *Prochlorococcus* MIT9313, which is a single-celled planktonic marine cyanobacterium [69]. A single LanM-type enzyme, ProcM, was found to cooperate with 29 different precursor peptides in vitro and in vivo. The enzyme thus showed a remarkable catalytic promiscuity. The precursor peptides were encoded either *cis* or *trans* to the ProcM enzyme, and the resulting family of lantipeptides was designated prochlorosins **18** [69].

### UV-absorbing pigments

**Figure 11:**
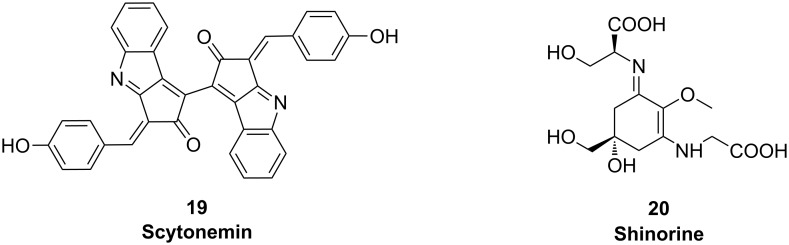
Structures of cyanobacterial sunscreen compounds.

Photosynthetic cyanobacteria depend on light for energy production. At the same time they are exposed to damaging solar UV radiation. Microorganisms have evolved different strategies to cope with UV light: UV avoidance/protection, and UV-absorption compounds. Cyanobacteria produce two types of sunscreen compounds, induced under UV irradiation: Scytonemin (**19**) and mycosporine-like amino acids **20** (Figure 11). Biosynthesis of the two groups of compounds has recently been elucidated, providing further examples for the fascinating natural product biochemistry of cyanobacteria.

#### Scytonemin

A gene cluster responsible for scytonemin (**19**) biosynthesis was initially discovered by random mutagenesis in the terrestrial symbiotic cyanobacterium *Nostoc punctiforme* [70]. The gene cluster contains a number of genes related to aromatic amino acid biosynthesis [70]. The biosynthetic route was proposed to start with tryptophan and tyrosine. Two of the initial steps of the sunscreen synthesis were reproduced in vitro [71]. The ORF NpR1275 was confirmed to act as a tryptophan dehydrogenase, whereas *p*-hydroxyphenylpyruvic acid was proposed to be generated by the putative prephenate dehydrogenase NpR1269. Both substrates are then further transformed by the thiamin diphosphate (ThDP)-dependent enzyme NpR1276 to isomeric acyloins representing one-half of the carbon framework of scytonemin [71]. The enzyme showed a remarkable selectivity for the specific C-C bond reaction that is unprecedented in natural systems. Further enzymatic transformations of the scytonemin pathway remain to be elucidated [71].

#### Mycosporine-like amino acids (MAAs)

Mycosporines were initially discovered in fungi and found to trigger light-induced fungal sporulation. Beside microsporines that consist of a single amino acid linked to cyclohexenone, cyanobacteria and other algae produce mycosporine-like amino acids (MAAs, **20**), which contain two substituents linked to the central ring by imine linkages. Four enzymes are involved in the synthesis of the specific MAA shinorine in *Anabaena variabilis* ATCC 29413: A dehydroquinase synthase homologue (DHQS), an *O*-methyl-transferase (O-MT), an ATP grasp ligase and an NRPS-like enzyme. Cloning of the entire gene cluster in *E. coli* led to the production of shinorine. The production of the intermediate 4-deoxygadusol by DHQS and O-MT could be reproduced in vitro. Unexpectedly, the DHQS homologue accepted sedoheptulose 7-phosphate as a substrate. The ATP grasp ligase and the NRPS-like enzyme could be related to the amino acid attachment to the cyclohexenone core by two unique enzyme strategies for imine formation.

## Conclusion

Genome sequencing projects of cyanobacteria have revealed a far greater potential of cyanobacteria to produce natural products than expected. It can thus be anticipated that genomic mining techniques that start with the theoretical prediction of structures from genomic data will be of increasing importance for the discovery of natural products in the future. In order to identify products of cryptic cyanobacterial pathways, several research groups have used heterologous expression approaches, e.g., for the discovery of cyanobactins, microviridins and prochlorosins. The expansion of the existing genomic mining toolbox to access the genomic resources and to discover hidden treasures remains a challenge for the future.

Cyanobacterial biosynthetic enzymes have revealed great potential for synthetic biology approaches for rational modifications of existing leading compounds and for the generation of libraries of new compounds. Enzymes catalyzing macrocyclization of cyanobactins, as an example, are highly promiscuous and have been successfully used for the cyclization of diverse peptides. Future studies will have to show how many of the fascinating biochemical features of cyanobacterial biosynthetic enzymes can be utilized for the design of novel compounds and their optimization toward medical targets.
